# AEON: Attractor Bifurcation Analysis of Parametrised Boolean Networks

**DOI:** 10.1007/978-3-030-53288-8_28

**Published:** 2020-06-13

**Authors:** Nikola Beneš, Luboš Brim, Jakub Kadlecaj, Samuel Pastva, David Šafránek

**Affiliations:** 8 Microsoft Research Lab, Redmond, WA USA; 9 University of Southern California, Los Angeles, CA USA; Faculty of Informatics, Masaryk University, Brno, Czech Republic

**Keywords:** Boolean networks, Attractors, Bifurcation analysis

## Abstract

Boolean networks (BNs) provide an effective modelling tool for various phenomena from science and engineering. Any long-term behaviour of a BN eventually converges to a so-called attractor. Depending on various logical parameters, the structure and quality of attractors can undergo a significant change, known as a bifurcation. We present a tool for analysing bifurcations in asynchronous parametrised Boolean networks. To fight the state-space and parameter-space explosion problem the tool uses a parallel semi-symbolic algorithm.

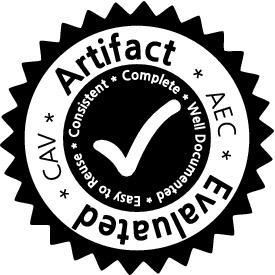



## Introduction

Boolean networks (BNs) provide an effective mathematical tool to model computational processes and other phenomena from science and engineering. BNs represent a generalisation of other relevant mathematical models, which appeared previously as cellular automata (CA), suggested by Wolfram [39] for computation modelling, or formal genetic nets [24] and Thomas networks [37], proposed for gene regulatory networks. This gives an idea of the versatility of BNs in different applications (mathematics, physics chemistry, biology, ecology, etc.) and engineering (computation, artificial intelligence, electronics, circuits, etc.).

The development of formal methods for analysis and synthesis of Boolean networks has recently attracted a lot of attention [11, 18, 20, 28, 36]. In this paper, we are primarily interested in BN models for computational systems biology [29]. In general, biological processes are emerging from complex inter- and intra-cellular interactions and they cannot be sufficiently understood and controlled without the help of powerful computer-aided modelling and analysis methods [38]. BNs serve an important purpose of describing overall interactions within a living cell at an appropriate level of abstraction and they provide a systematic approach to model crucial states of cell dynamics – so-called *phenotypes* [22].

The level of abstraction provided by BNs makes them an important tool for design of targetted therapeutic procedures such as cell reprogramming [36] based on changing one cell phenotype to another, allowing regeneration of tissues or neurons [21]. Since phenotypes are determined by long-term behaviour of biological systems, fully automatised identification of phenotypes by employing BN models is a necessary step towards the future of modern medicine. Owing to the fact there is a continuous lack of sufficiently detailed (mechanistic) information on biological processes, there is definitely a need to work with models involving uncertain (or insufficient) knowledge. In this paper, we present a unique tool that makes a significant contribution towards fully automatised analysis of long-term behaviour of BN models with uncertain knowledge.

We start with giving some intuition on BNs. A BN consists of a set of Boolean *variables* whose state is determined by other variables in the network through a set of Boolean *update functions* assigned to the variables (different update functions can be assigned to different variables) and *regulations* placed on them. If at each point of time all the update functions are applied simultaneously we speak about *synchronous dynamics*, if only one of the update functions is chosen non-deterministically to modify the corresponding Boolean variable, we speak of *asynchronous dynamics*. In this paper we consider asynchronous Boolean networks only.

In real-world applications, the update functions for some of the variables are typically (partially) unknown and are represented as logical *parameters* of the network. We speak of *parametrised Boolean networks* [40] in this case. If all the parameters are fixed to a concrete Boolean function, a parametrised BN turns into a (non-parametrised) BN.

The long-term behaviour of a BN, starting from an initial state, has three possible outcomes. Briefly, the first situation is when the network evolves to a single stable state. Such states are the fixed points or *point attractors* or *stable states*. The second situation is that the network periodically oscillates through a finite sequence of states—an *oscillating attractor* or *attractive cycle* (the discrete equivalent of a limit cycle in continuous systems). The third case is what we call a *disordered attractor* (or chaotic oscillation [32]), an attractor that is neither stable not periodically oscillating and in which the system may behave unpredictably, due to the nondeterminism of the asynchronous semantics of BNs. Attractors are particularly relevant in the context of biological modelling as they are used to represent differentiated cellular types or tissues (in the case of fixed points) [2] and biological rhythms or oscillations (in the case of cycles) [17].

The set of network states that converge to the same attractor forms the *basin of attraction* of that attractor [7]. Attractors (and their basins) are disjoint entities and the state space is compartmentalised by imaginary “attractor boundaries”. The entire dynamics of a Boolean network can be represented as a state transition system in which the trajectories from initial states are depicted, revealing the basins of attraction and associated attractors. We call such a representation the *attractor landscape* of the network [13].

In parametrised BNs the attractor landscape changes as the parameters are varied. Some of these changes may lead to a qualitatively different landscape (defined, e.g., in the count and/or quality of attractors). Such a qualitative change is called a *bifurcation* and the values of parameters for which it occurs are called *bifurcation points*. Determining (all) bifurcation points for a network, called *attractor bifurcation analysis*, is an important task in the analysis of BNs [4].

While BN models are intuitive, mathematically simple to describe, and supported by analytical methods [12], analysis of large models appearing in real cases is severely limited by the lack of robust computational tools running efficiently on high-performance hardware. Several computational tools have been developed for construction, visualisation and analysis of attractors in non-parametrised BNs. Amongst them, the established tools include ATLANTIS [34], Bio Model Analyzer (BMA) [6], BoolNet [31], PyBoolNet [27], lnet [7], The Cell Collective [23], CellNetAnalyzer [25], and ASSA-PBN [30]. Another group of existing tools targets the parameter synthesis problem for parametrised BNs. The most prominent tools here are GRNMC [20], GINsim [10] (indirectly through NuSMV [14]), and TREMPPI [35]. In general, parameter synthesis tools can be used to identify parameters producing a specified long-term behaviour (depending on the logics employed), however, they do not provide a sufficient solution for identification and classification of all attractors in the system. Finally, it is worth noting that there have recently appeared several tools aiming at control of cell behaviour through BNs (i.e., driving a cell into the desired state). A well-known representative of these tools is ViSiBooL [33].

To the best of our knowledge, none of the existing tools is capable of performing attractor bifurcation analysis in parametrised models. Bifurcation analysis has been recently recognised as a fundamental approach that provides a new framework for understanding the behaviour of biological networks. The ability to make a dramatic change in system behaviour is often essential to organism function, and bifurcations are therefore ubiquitous in biological networks such as the switches of the cell cycle. The tool AEON is supposed to fill in the gap in the existing tools supporting analysis of Boolean network models.

AEON builds on methods and algorithms for asynchronous parametrised BNs we have introduced in our previous research [1, 3–5]. To deal with the state-space and parameter-space explosion problem, the tool implements a shared-memory parallel semi-symbolic algorithm. The results the tool provides to the user can be used for example to the design of “wet” experiments, better understanding of the system’s dynamics, or to control or re-program the system. As attractors model phenotypes, one of the most urgent needs for computer aided support, such as AEON can provide, is in applications in therapeutic innovations.

We believe that attractor bifurcation computed by AEON will shift the current technology toward a comprehensive method when integrated with tools aimed at control or other analysis methods.

## Attractors in Parametrised Boolean Networks

In this section, we define precisely the problem of attractor bifurcation analysis. We also give an overview of the necessary technical background needed to describe the algorithmic solution and its implementation. More details can be found in [4].

A *Boolean network (BN)* consists of a finite set of *state variables* $\mathcal {V}$ (whose elements we denote by $\mathtt {A}$, $\mathtt {B}$, ...), a set of *regulations* $R \subseteq \mathcal {V}\times \mathcal {V}$, and a family of Boolean *update functions* $\mathcal F = \{ F_{\mathtt {A}} \mid \mathtt {A} \in \mathcal {V}\}$. If $(\mathtt {B}, \mathtt {A}) \in R$, we say that $\mathtt {B}$ is a *regulator* of $\mathtt {A}$. For each $\mathtt {A} \in \mathcal {V}$, we call the set $\mathcal {C}(\mathtt {A}) = \{\mathtt {B} \in \mathcal {V}\mid (\mathtt {B}, \mathtt {A}) \in R \}$ of its regulators the *context* of $\mathtt {A}$. A *state* of the BN is an assignment of Boolean values to the variables, i.e. a function $\mathcal {V}\rightarrow \{0, 1\}$. The type signature of each update function $F_{\mathtt {A}}$ is given by the context of $\mathtt {A}$ as $F_{\mathtt {A}}: \{0, 1\}^{\mathcal {C}(\mathtt {A})} \rightarrow \{0, 1\}$.

In Boolean networks, one often describes various properties of the network regulations. Here, we focus on three most basic types of regulation: We say that $(\mathtt {A}, \mathtt {B}) \in R$ is *observable* if there exists a state where changing the value of $\mathtt {A}$ also changes the value of $F_{\mathtt {B}}$. In the tool, edges that might be non-observable are drawn using dashed lines.

We say that a regulation $(\mathtt {A}, \mathtt {B}) \in R$ is *activating* if by increasing $\mathtt {A}$, one cannot decrease the value of $F_{\mathtt {B}}$. Symmetrically, the regulation is *inhibiting* if by increasing $\mathtt {A}$, one cannot increase the value of $F_{\mathtt {B}}$. In the tool, activating edges are denoted using green colour and sharp arrow tips, inhibiting edges are denoted using red colour and flat arrow tips, and edges that might be neither activating nor inhibiting are denoted using grey colour.

**Fig. 1.**
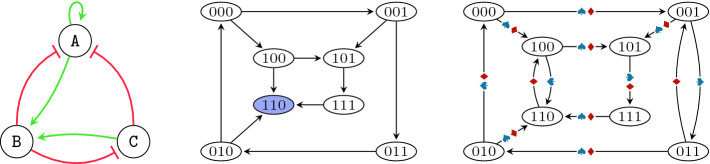
Illustration of a (parametrised) BN and its state transition graph. (Color figure online)

Let us now consider an example of a BN with $\mathcal {V}= \{\mathtt {A}, \mathtt {B}, \mathtt {C}\}$, the regulations *R* as denoted in Fig. 1 (left) and the update functions: $F_{\mathtt {A}} = \mathtt {A} \vee \lnot \mathtt {B} \vee \lnot \mathtt {C}$, $F_{\mathtt {B}} = \mathtt {A} \vee \mathtt {C}$, $F_{\mathtt {C}} = \lnot \mathtt {B}$. We can see that all regulations are observable and the colour (and shape) of the arrows respects the properties of activation and inhibition, e.g. $(\mathtt {B}, \mathtt {A})$ is an inhibition, because by increasing the value of $\mathtt {B}$, we cannot increase the value of $F_{\mathtt {A}}$.

The semantics of a Boolean network is given as a directed *state transition graph*. The state space of the graph is the set of all possible assignments of Boolean values to the variables, i.e. $\{0, 1\}^\mathcal {V}$. We consider the state of the Boolean network to evolve in an *asynchronous* manner, i.e. each variable is updated independently. We thus add a transition $s \rightarrow t$ if $s \ne t$ and if there exists a variable $\mathtt {A}$ such that $t(\mathtt {A}) = F_{\mathtt {A}}(s)$ and $t(\mathtt {X}) = s(\mathtt {X})$ for all $\mathtt {X} \in \mathcal {V}\setminus \{\mathtt {A}\}$. We also use the notation $\rightarrow ^*$ to denote the reflexive and transitive closure of $\rightarrow$, i.e. $s \rightarrow ^* t$ means that the state *t* is reachable from the state *s*.

The semantics of the BN from our example is illustrated in Fig. 1 (middle). The states are represented as Boolean triples denoting the values assigned to the variables $\mathtt {A}$, $\mathtt {B}$, and $\mathtt {C}$, respectively.

The long-term behaviour that we are interested in is captured by the notion of *attractors*. In discrete-state systems, attractors are represented by terminal strongly connected components (TSCCs) of the graph. A TSCC is a maximal set of states *S* such that for all *s*, $t \in S$, $s \rightarrow ^* t$, and for all $s \in S$, $s \rightarrow t$ implies $t \in S$.

To classify the attractors of a given BN, we consider three primary kinds of long-term behaviour:

- *stability* ($\odot$) We say that an attractor is stable, if it consists of a single state, in which the network stays forever.
- *oscillation* ($\circlearrowleft$) We consider an attractor to be oscillating if it is a single cycle of states. The size of such cyclic attractor is often referred to as its *period*.
- *disorder* ($\rightleftarrows$) Finally, an attractor is said to be disordered if it is neither stable nor oscillating. This means that although the network will stay in the attractor forever, it will behave somewhat unpredictably due to nondeterminism.

The long-term behaviour of a BN is then characterised by a multi-set over the universe of the three behaviours $\{ \odot , \circlearrowleft , \rightleftarrows \}$. We call such multi-set a *behaviour class* and we denote the set of all possible behaviour classes $\mathfrak {C}$. In our example, the BN has only one attractor, and this attractor is stable; it consists of the single state 110, see Fig. 1 (middle).

To deal with the fact that the update function family $\mathcal {F}$ might not be fully known, we extend the Boolean network with a set of *logical parameters* which determine the exact behaviour of each update function. These parameters have the form of uninterpreted Boolean functions, which can be used as part of the update functions’ description.

Formally, we assume a finite set of *parameter names* $\mathfrak {P}$, whose elements we denote by $\mathtt {P}$, $\mathtt {Q}$, ...; we assume that every $\mathtt {P} \in \mathfrak {P}$ has an associated arity $a_\mathtt {P}$ meaning that $\mathtt {P}$ is an $a_\mathtt {P}$-ary uninterpreted function over Boolean values. Note that nullary uninterpreted functions are also allowed and can be seen as simply Boolean parameters. We call an interpretation that assigns to each $\mathtt {P} \in \mathfrak {P}$ an $a_\mathtt {P}$-ary Boolean function a *parametrisation*. We usually work with a subset of parametrisations, called the *valid* parametrisations and denoted by *P*.

A *parametrised Boolean network* consists of a set of variables $\mathcal {V}$, a set of regulations $R \subseteq \mathcal {V}\times \mathcal {V}$ as in the non-parametrised case, a set of parameter names $\mathfrak {P}$, its associated set of valid parametrisations *P*, and a family of *parametrised update functions* $\mathfrak {F} = \{\widehat{F}_\mathtt {A}\mid \mathtt {A} \in \mathcal {V}\}$. Each $\widehat{F}_\mathtt {A}$ is written as a Boolean expression that may contain the uninterpreted functions of $\mathfrak {P}$.

Let us now modify the previous example so that we view the BN from Fig. 1 (left) as a parametrised one with the following update functions: $\widehat{F}_\mathtt {A} = \mathtt {A} \vee \lnot \mathtt {B} \vee \lnot \mathtt {C}$, $\widehat{F}_\mathtt {B} = \mathtt {P}(\mathtt {A}, \mathtt {C})$, $\widehat{F}_\mathtt {C} = \lnot \mathtt {B}$, where $\mathtt {P}$ is a parameter name with arity 2. The set of valid parametrisations is constrained symbolically using the description of activations and inhibitions in Fig. 1 (left). In this case, there are only two possible parametrisations $p_1$ (denoted by  ) and $p_2$ (denoted by  ). The parametrisation $p_1$ assigns to $\mathtt {P}$ the function $(x, y) \mapsto x \vee y$, while $p_2$ assigns to $\mathtt {P}$ the function $(x, y) \mapsto x \wedge y$. Note that other assignments would violate the description, namely that both $(\mathtt {A}, \mathtt {B})$ and $(\mathtt {C}, \mathtt {B})$ are observable and activating.

By fixing a concrete parametrisation $p \in P$, we can interpret all the parameter names and thus transform the parametrised update functions into non-parametrised ones, obtaining a (non-parametrised) BN, called the *p-instantiation* of the parametrised BN. We then generalise the definition of attractors to parametrised BNs, saying that a set of states *S* is an *attractor in parametrisation* $p \in P$ if *S* is an attractor in the *p*-instantiation.

The asynchronous semantics of a parametrised BN can be described using an *edge-coloured* state transition graph. The transitions of this graph are assigned a set of so-called *colours*—in our case, the colours correspond exactly to the parametrisations. The states are given as in the non-parametrised case. We then say that $s \rightarrow t$ if there exists a parametrisation *p* such that $s \rightarrow t$ in the *p*-instantiation. The set of colours of $s \rightarrow t$ is the set of all such parametrisations. In our example, the graph is depicted in Fig. 1 (right; the edges are annotated with  ,  , or both).

**Problem Formulation.** We now formulate the problem of *attractor bifurcation analysis of parametrised BN* as follows: Given a parametrised BN with a set of valid parametrisations *P*, compute the *bifurcation function* $\mathcal A: P \rightarrow \mathfrak C$ that assigns to each parametrisation *p* the behaviour class of the *p*-instantiation of the given parametrised BN.

In our example, the function $\mathcal A$ maps $p_1$  to $\{\odot \}$ (one stable attractor $\{110\}$) and $p_2$  to $\{\circlearrowleft \}$ (one oscillating attractor $\{100, 101, 111, 110\}$).

## Attractor Bifurcation Analysis with AEON

**Fig. 2.**
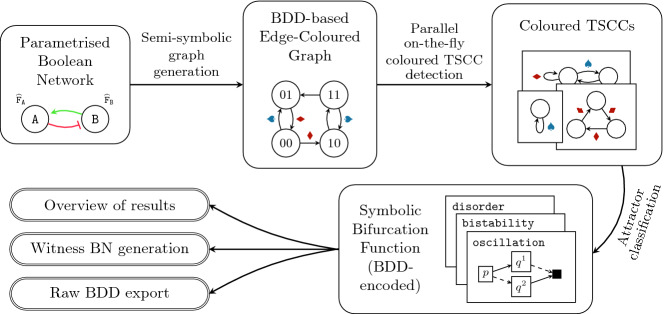
The workflow of the AEON tool.

The workflow of our approach, as implemented in the tool, is illustrated in Fig. 2. As an input, we take a parametrised BN including a graphical description of the regulations. The tool computes its asynchronous semantics as a symbolic edge-coloured graph represented using BDDs [8]. This is then used as an input to a parallel TSCC detecting algorithm based on [1], which extracts the attractors on the fly. Each attractor is classified as one of the three above-mentioned types and this information is used to incrementally build the bifurcation function $\mathcal A$, also represented symbolically using BDDs. More details about the algorithm as well as the classification procedure can be found in [4].

**Fig. 3.**
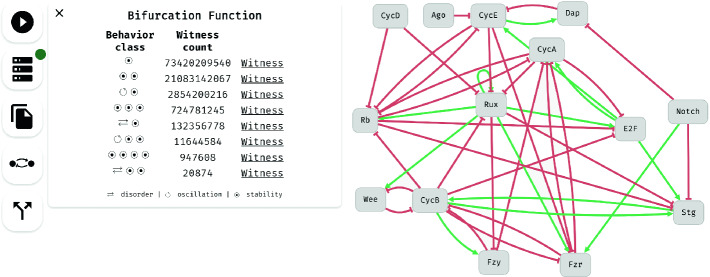
Screenshot of the tool displaying a parametrised BN together with the bifurcation analysis results.

The bifurcation function induces a partitioning of the parameter space in which two parametrisations are equivalent if their *p*-instantiations have the same behaviour class. This partitioning is presented to the user as a list of behaviour classes together with the cardinality of the respective parameter space partitions, see Fig. 3. The user can select one of these classes and obtain a *witness BN*, i.e. a *p*-instantiation of the parametrised BN where *p* is one of the corresponding parametrisations. Finally, the tool also provides the whole bifurcation function encoded as BDDs—this output can be used for post-processing by further tools.

## Implementation

**Fig. 4.**
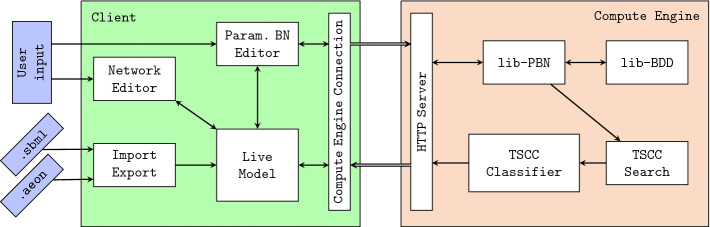
Overview of the tool architecture showing the main components of the GUI client and the compute engine. Arrows represent the general flow of information between individual components.

The tool architecture consists of two components as seen in Fig. 4: the *compute engine*, and a web-based, user-facing GUI application (*the client*). The engine is responsible for the actual computation and acts as a web server to which the client establishes a connection. Using web-based GUI enables portability across different platforms, and the separation of the user interface from the compute engine enables the user to run the computation remotely on high-performance hardware.

One of the responsibilities of the client is to provide a user friendly, multi-platform editor of parametrised BNs, since no popular BN editors currently support parameters. Architecturally, the client consists of several modules:

- Live Model: In-memory representation of the currently displayed model.
- Compute Engine Connection maintains the communication between the client and the compute engine.
- Network Editor: An interactive drag-and-drop editor for drawing the structure of the BN (variables, regulations). The implementation is based on the popular Cytoscape [19] library for graph visualisation and manipulation.
- Parametrised BN Editor: The update functions can be modified in a separate parametrised BN editor tab. This module is also responsible for basic integrity checks and static analysis of the BN, some of which is asynchronously deferred to the compute engine.
- Import/Export facilitates serialisation and transfer of the BNs to other tools. We currently provide a compact text-based format specifically designed for AEON and a universally adopted XML-based SBML level 3 qual standard [9].

The compute engine is written entirely in Rust to ensure fast and reliable operation (as well as easy portability). The functionality of the engine is split into separate libraries to allow later reuse:

- lib-BDD: Our own robust, thread-safe, scalable Rust-based implementation of BDDs.
- lib-PBN: A general purpose library for working with parametrised BNs. It provides serialisation to and from the AEON text format as well as SBML. Most importantly, it provides a parameter encoder that maps sets of parametrisations of the parametrised BN to BDDs. Using this encoder, the library implements an on-the-fly generation of the edge-coloured state transition graph corresponding to the asynchronous semantics of the given parametrised BN.
- TSCC Search algorithm implements the component search algorithm as presented in [1]. The algorithm uses parallel reachability procedures as well as asynchronous processing of independent parts of the state space to fully utilise available CPUs and thus speed up the computation. The algorithm is extended with appropriate cancellation points so that the user can stop the computation when needed.
- TSCC Classifier classifies and stores information about the discovered components. Specifically, for each non-empty behaviour class, we store a BDD representation of the parametrisations that result in this type of behaviour.

Aside from the general overview of the tool, we would like to highlight two additional aspects of AEON:

*On-the-Fly Results:* The attractors are discovered gradually. At any time during the computation the user may inspect the partial result, i.e. the bifurcation function computed so far. Although this is not the final outcome, such partial information can still prove useful, e.g. if unexpected attractor behaviour is found and the update functions of the model need to be adjusted.

*SBML with Parameters:* In our implementation, when dealing with fully instantiated networks, we always output valid SBML. Unfortunately, the current SBML standard does not allow parameters or uninterpreted functions inside the update function terms. In fact, the update functions in SBML are represented using MathML^1^ which in general allows arbitrary mathematical expressions, but its use in SBML is restricted. To export parametrised BNs, we intentionally disregard the restriction and our tool produces MathML formulae with parameters. Note that existing SBML implementations can be easily extended to also support parametrised BNs, since they already contain MathML parsers.

Both the client^2^ and the compute engine^3^ are released as open source under the MIT License. Furthermore, an online version of the client is available at https://biodivine.fi.muni.cz/aeon/, including links to pre-built binaries of the computation engine for all major OSes.

## Evaluation

We evaluated the efficiency and applicability of AEON tool on a set of real biological models taken from the GINsim model database [10], ranging from small toy examples to large real world models. The experiments were performed on a 32-core AMD Ryzen workstation with 64 GB of memory. All tested models are available in AEON source code repository (see footnote 3) as benchmark models.

The results are reported in Table 1. In general, the results show that the combination of symbolic representation of parametrisations and shared-memory parallel exploration of the state space allowed us to handle realistic BNs with large parameter spaces and non-trivial number of attractor bifurcations in reasonable time. Finally, let us note that the findings provided by AEON are in line with known properties of these biological models and even have a potential to provide new insights on the modelled biological processes.

**Table 1.** The evaluation results. Number of classes refers to the number of distinct behaviour classes discovered by the algorithm. The times in the form minutes:seconds refer to total runtime on 1 and two 32 CPU cores respectively.

Model name	State space size	Param. space size	No. of classes	Time (1cpu)	Time (32cpu)
Asymmetric Cell Division	$2^{5}$	$\sim$2$^{18}$	11	0:05.62	0:03.39
Budding Yeast (Orlando)	$2^{9}$	$\sim$2$^{18}$	6	0:35.22	0:02.93
TCR Signalisation	$2^{10}$	$\sim$2$^{14}$	17	0:26.61	0:04.42
Drosophila Cell Cycle	$2^{14}$	$\sim$2$^{36}$	8	27:48.1	1:42.28
Fission Yeast Cell Cycle	$2^{10}$	$\sim$2$^{31}$	201	25:20.9	4:00.29
Mammalian Cell Cycle	$2^{10}$	$\sim$2$^{44}$	176	38:39.6	8:02.14
Budding Yeast (Irons)	$2^{18}$	$\sim$2$^{26}$	7	Timeout	52:28.1

In particular, in the case of the *TCR Signalisation* model, the authors have shown in [26] that their non-parametrised model produces seven possible stable states and one non-trivial attractor. By using AEON, we were able to confirm their findings as well as analyse a fully parametrised version of the model, finding sixteen other possible behaviours. Interestingly, in this model, all discovered seventeen behaviour classes consist of exactly eight attractors.

For the *Budding Yeast (Orlando)* model [16], the authors state that for several different parametrisations, the model always reaches a stable state (based on simulation). Our analysis performed with AEON has confirmed that the original instantiation of the model has indeed a single stable attractor. Moreover, we have found that in the fully parametrised version of the model, almost ninety thousand instantiations have a single stable attractor. Additionally, we have also found there is almost an equal number of instantiations producing disordered attractors and also several oscillating attractors. AEON is capable to generate witnesses for all of these situations thus opening the biological questions targeting the existence of the corresponding phenotypes in nature.

The *Fission Yeast Cell Cycle* model [15] is known to contain one primary stable attractor as well as eleven artificial attractors. It is known that various multi-valued modifications of the original model exist that remove these artificial stable attractors from the model while preserving the only single stable attractor [16]. By parametrising the model adequately and applying our method using AEON, we have discovered that a large portion of the parameter space of the model also produces a single stable attractor.

## Footnotes

1 https://www.w3.org/TR/MathML3/.

2 https://github.com/sybila/biodivine-aeon-client.

3 https://github.com/sybila/biodivine-aeon-server.
